# Biting the hand that feeds: current opinion on the interpersonal causes, correlates, and consequences of borderline personality disorder

**DOI:** 10.12688/f1000research.9392.1

**Published:** 2016-11-30

**Authors:** Sheila E. Crowell

**Affiliations:** 1 Department of Psychology, University of Utah, 380 South 1530 East, Room 502, Salt Lake City, UT, 84112, USA

**Keywords:** Borderline personality disorder, BPD, personality disorders, interpersonal causes

## Abstract

Borderline personality disorder (BPD) is a complex psychiatric diagnosis characterized by dysregulated behaviors, emotions, cognitions, and interpersonal relationships. In recent years, developmental psychopathologists have sought to identify early origins of BPD, with the ultimate goal of developing and providing effective preventative interventions for those at highest risk. In addition to heritable biological sensitivities, many scholars assert that environmental and interpersonal risk factors contribute to the emergence and maintenance of key borderline traits. Nonetheless, many BPD researchers examine only affected individuals, neglecting the family, peer, couple, and other dynamic contextual forces that impinge upon individual-level behavior. In the past decade, however, theoretical and empirical research has increasingly explored the interpersonal causes, correlates, and consequences of BPD. Such work has resulted in novel research and clinical theories intended to better understand and improve interpersonal dynamics among those with borderline traits. A major objective for the field is to better characterize how interpersonal dynamics affect (and are affected by) the behaviors, emotions, and thoughts of vulnerable individuals to either reduce or heighten risk for BPD.

## Introduction

Humans are a social species, and, as such, interpersonal relationships are critical to health, wellbeing, and survival ^1– 4^. Such connections begin *in utero*, continue with caregiver–child attachments, and are reinforced through peer relationships, sexual partnerships, and pair bonding. Accordingly, most conceptualizations of personality and psychopathology discuss social mechanisms of risk/resilience ^5– 13^, and interpersonal theories are central to several prominent psychotherapy models ^14– 20^. The biosocial theory of borderline personality disorder (BPD) is one example of an etiological model that highlights contextual risk factors as contributing to the emergence and maintenance of borderline traits ^14, 21^. According to this theory, impulsive and emotionally sensitive youth are at higher risk for BPD when exposed to social environments characterized by emotional invalidation and intermittent reinforcement/punishment of emotional volatility, overcontrol, or both ^22^. Following from this model, early detection and prevention of BPD requires identifying emotionally sensitive youth who are living in environments characterized by high stress, emotional invalidation, and/or conflict. Once BPD traits emerge and begin to stabilize, interventions could potentially be enhanced by including friends, family members, and romantic partners in the conceptualization and treatment of BPD ^23– 26^.

The idea that interpersonal distress can be both cause and consequence of BPD is neither novel nor controversial ^27^. In a landmark text on interpersonal aspects of BPD, Benjamin ^19^ describes one type of patient as having “unpredictably invasive and traumatic abandonment experiences…[which] set the prototype for the interactional patterns so often seen between the [patient] and the health care provider” (page 9). Although trauma and abandonment are no longer considered to be central to the etiology of BPD, those with the diagnosis often have a distressing interpersonal history characterized by loss, socioeconomic disadvantage, or poor fit with the caregiving environment ^14^. As a result, many individuals with BPD struggle to form healthy relationship patterns with important people in their lives, such as their therapist. Thus, it is well established that healthy relationships are foundational to emotional and physical health *and* they are highly disrupted in BPD, leading to further distress. In light of this, it is surprising that interpersonal contributors to borderline traits are still relatively neglected in a literature that clearly needs to examine relationships with more sophisticated research designs and statistical methods, such as daily diary studies and dynamical systems approaches ^28– 34^. Similarly, the next wave of prevention and intervention studies must (1) move some aspects of treatment out of the therapy room and into daily life, (2) incorporate friends and family members into treatment, and (3) increasingly integrate the science of relationships into psychiatric and other biological interventions.

## Interpersonal causes of BPD

Psychopathology is complex, and there is no single causal process leading to BPD. Nonetheless, there are identifiable precursors and risk factors that contribute to the emergence and maintenance of borderline traits ^35– 40^. Above all, BPD is a disorder of instability—in self-concept, emotions, behaviors, and relationships ^41, 42^. Leading theorists hypothesize that this instability develops through inconsistency in early attachment relationships, leading to lack of trust in caregivers to serve as co-regulators of emotions and behaviors ^43– 49^. At one extreme, physical or sexual abuse by a trusted caregiver appears to be associated with BPD traits, especially self-harm and suicidality ^50– 55^ (however, see also 56, 57). At the other extreme, a relatively benign mismatch between parenting style and child temperament may increase risk for BPD traits *if* the child has heritable vulnerabilities that render him/her uniquely sensitive to the caregiving environment ^6, 12, 26, 58– 60^. Importantly, the parenting interventions for both extremes are highly similar: reduce blame, minimize stigma, and also provide support, skills coaching, and strategies for caregivers ^61, 62^. From a research perspective, there are still relatively few prospective studies that examine early parenting and the emergence of BPD ^63, 64^. In particular, there is very little research examining BPD-specific outcomes in longitudinal studies of parent–child play, parenting strengths, behavioral parenting interventions, early childhood education, and other factors that should reduce risk for BPD. Clinically, given that many BPD traits are partially heritable (e.g. impulsivity and emotional sensitivity), early interventions may have benefits for parents while also reducing intergenerational transmission of psychological distress.

## Interpersonal correlates of BPD

There is a growing literature examining interpersonal correlates of BPD in adolescents and adults. Studies find that adults with borderline traits have disrupted social networks ^65^, impaired couple relationships ^66^, social anxiety ^67^, and difficulties trusting others ^68^ and also struggle to form attachments with and manage their own children ^69– 75^. Adolescents with borderline traits experience peer problems ^76^, parent–child relationship struggles ^22, 77^, and sexual “compulsivity” ^78^ and have problems with romantic partners ^79^. In spite of this, there are no randomized controlled trials testing the effectiveness of adjunctive couple’s therapy among partnered adults with BPD ^80^. Similarly, there are only a few clearly articulated protocols for parent/family involvement in the treatment of BPD or of youth with borderline traits ^81^, leading practitioners to meet this need in a variety of ways that are often not yet empirically validated or covered by third-party payers (e.g. adolescent and family skills group, parent coaching, or individual parenting therapy for the parent). Basic science researchers also rarely bring friends or family members into the lab, even though relationship quality is known to affect prognosis ^82^. Thus, there is an urgent need to understand dynamic interaction patterns between those with borderline traits and their loved ones ^83^. For example, it is unknown which parent/friend/spouse behaviors are experienced as supportive or soothing to those with BPD. Similarly, it is unclear which interpersonal skills or strategies would be most helpful for fostering lasting resilience in relationships. The fact that most research is conducted at the individual level of analysis perpetuates treatment strategies targeted at the individual, rather than the system. This communicates a powerful message of blame, when in fact many problems may exist within dynamic interaction patterns *between* persons rather than *within* any one person.

## Interpersonal consequences of BPD

There is little doubt that those with BPD have a powerful effect on those around them. Borderline traits are highly evocative, and these traits can be painful for friends and family members, although research exploring this topic is limited. For example, abandonment fears of those with BPD could lead loved ones to forego other activities, which could shrink the social networks of loved ones. Anger, fear, hopelessness, rejection sensitivity, reassurance seeking, experiential avoidance, interpersonal aggression, and emotional lability are correlates of borderline traits ^84– 93^, and these features could contribute to partner selection, relationship conflict, and increased interpersonal distress over time. Self-injury and suicide are behaviors that could also have a lasting effect on friends and family members ^94– 96^. Parents with BPD may also affect offspring, with lasting consequences for child emotion regulation capacity ^69, 74^. Unfortunately, the existing studies focus almost exclusively on negative interpersonal effects of those with BPD and, to my knowledge, there are no studies that ask what people love and enjoy about their family member with BPD. Few researchers have considered the potential adaptive features of the disorder ^97^, in spite of theories that other diagnoses, such as depression ^98^ and attention deficit hyperactivity disorder, may actually have evolutionary advantages ^99^. Thus, with few exceptions ^100^, the existing literature is skewed heavily toward identifying areas of dysfunction within the individual and early caregiving environment while neglecting areas of strength among those with BPD and their interpersonal networks.

## Summary and future directions

In sum, BPD is a complex psychiatric disorder with causes, correlates, and consequences that are multidetermined ^101^. Although there is a rich theoretical literature on interpersonal factors and BPD, the empirical literature lags far behind the theorized contextual mechanisms of risk. For example, although there are some prospective studies linking childhood physical and sexual abuse with later BPD, relatively few studies examine *why* neglect or abuse predicts BPD in some cases. Thus, longitudinal mediation studies are needed to examine cumulative changes in the social (e.g. trust and co-regulation) and neurobiological (e.g. frontolimbic and neurotransmitter functioning) processes that link adverse childhood experiences with borderline pathology and other diagnoses ^43, 86^. There is a similarly small literature examining the question of *for whom* do these high-risk environments contribute to BPD and which children are relatively resilient even in the face of extreme adversity ^49, 86^. Researchers studying BPD could draw upon the research designs and methods within the broader developmental psychology and developmental psychopathology literatures. Extending research on risk and resilience to the development of BPD is an important future direction and will involve identifying both mediators and moderators of high-risk developmental trajectories. Based upon developmental research into other forms of psychopathology, it is likely that risk for BPD will not be tied to any single event or stressor. Rather, borderline traits are likely to emerge slowly owing to an accumulation of problematic interactions among vulnerable individuals, their caregivers/peers, and a risky developmental context.

There are many challenges facing BPD researchers and clinicians ^35, 102^. For example, there is a growing literature examining biological factors associated with BPD but the translation to validated medical or psychopharmacological approaches has been slow ^103– 105^. To date, there are no medications approved specifically for use in BPD, and intensive psychotherapy (usually lasting 12 months or more) is the treatment of choice for affected individuals ^106, 107^. Such interventions are expensive and time intensive and do not reduce all BPD symptoms for all affected individuals. Thus, even though there have been tremendous advances in psychotherapy and pharmacotherapy for those with BPD, it is increasingly clear that early intervention and prevention could be the most economical way to reduce the financial and emotional costs associated with a borderline diagnosis. As a first step, both researchers and clinicians need to extend their work outside of the laboratory/therapy room and into the daily lives of those with BPD. In dialectical behavior therapy, a leading treatment for BPD, the therapist provides phone-based coaching to enhance skills generalization from the therapy context into real world settings ^108^. However, there is little research examining the incremental benefit of skills coaching over other elements of therapy or relative to multiple weekly therapy sessions. To my knowledge, there is no research examining coordination with schools, churches, or other community agencies as a regular component of BPD prevention or intervention.

As a second step, therapists should operate under the assumption that relationships are affected by and are affecting the person with BPD. Indeed, current work on the social neuroscience of emotion regulation suggests that humans are neurobiologically “wired” to be embedded in social networks and show emotional distress when social connection is lacking ^109– 111^. Following on from this assumption, it is unlikely for a person to attain full remission if the dynamic interaction patterns that shape problematic behaviors remain unchanged. Thus, it may be necessary for BPD treatments to involve parents, friends, significant others, and family members in treatment. Unfortunately, there are very few evidence-based models for how to structure these sessions, and structure is essential for work with a dysregulated individual who is being actively exposed to interpersonal cues for maladaptive behavior. Researchers could also extend work outside of the laboratory to better understand social regulation of emotion in daily life. Daily diary methods and wearable psychophysiological monitoring devices could provide useful information on the factors that maintain conflict, risk-taking behaviors, hopelessness, or suicidality.

Finally, medical providers and those providing pharmacological treatments should also consider the importance of relationships and stability in the lives of those with BPD ^27^. There is enormous pressure on medication providers to help stabilize and resolve the intense emotional lability experienced by those with BPD. Often this pressure comes from patients, who may experience their medications as effective one day and ineffective another or who may misuse or discontinue medications when the benefits are unclear. Pressure may also come from family members or third-party payers who often hope for quicker or more lasting benefits from psychotropic medications. Research on the developmental neuroscience of BPD suggests that the emotional lability seen in BPD is partially shaped and maintained within social environments ^102^. Thus, although there are clearly heritable biological vulnerabilities, especially for those that underlie impulsive behaviors, medications alone are unlikely to resolve BPD symptoms if there is ongoing instability in relationships, sleep, or eating or if there are a high number of daily stressors. Similar to most psychiatric disorders, the combination of medication and therapy will likely be essential for treatment success ^112^.

In conclusion, current research on BPD suggests that interpersonal factors contribute to the emergence and maintenance of the disorder, but there are many unanswered questions. Specifically, most of the research examines a small number of stressors (e.g. reports of abuse) rather than intensive daily measurements of dynamic contextual forces in the lives of those with BPD. The data that do exist are skewed toward a negative perception of those with BPD and their loved ones. Meanwhile protective factors that promote resiliency and healing are underexplored, both in research and in treatment. Treatments focused on skills for validation and enhancing positive relationship factors could represent a novel direction for treatment development. Unfortunately, in spite of a large and growing body of research articulating the importance of social relationships for promoting health and wellbeing, there is a lack of evidence-based protocols for involving friends and family members in BPD-focused interventions. The treatment of personality disorders is also neglected by many third-party payers, who rarely cover a full year of intensive treatment and almost never cover skills groups, couple’s therapy, or family therapy. Without additional research, treatment development, translation to clinical practice, and advocacy to insurance companies, individuals with BPD may continue to struggle in relationships—metaphorically “biting the hand that feeds them”—with few skills and supports for fostering the types of social connections that contribute to health and wellbeing.
